# Budding of Tiger Frog Virus (an Iridovirus) from HepG2 Cells via Three Ways Recruits the ESCRT Pathway

**DOI:** 10.1038/srep26581

**Published:** 2016-05-26

**Authors:** Shu Mi, Xiao-Wei Qin, Yi-Fan Lin, Jian He, Nan-Nan Chen, Chang Liu, Shao-Ping Weng, Jian-Guo He, Chang-Jun Guo

**Affiliations:** 1 Guangdong Provincial Key Laboratory of Marine Resources and Coastal Engineering/South China Sea Bio-Resource Exploitation and Utilization Collaborative Innovation Center, School of Marine, Sun Yat-sen University, 135 Xingang Road West, Guangzhou 510275, P. R. China; 2 MOE Key Laboratory of Aquatic Product Safety/State Key Laboratory for Biocontrol, School of Life Sciences, Sun Yat-sen University, 135 Xingang Road West, Guangzhou 510275, P. R. China; 3 Institute of Aquatic Economic Animals and Guangdong Province Key Laboratory for Aquatic Economic Animals, Sun Yat-sen University, 135 Xingang Road West, Guangzhou 510275, P. R. China.

## Abstract

The cellular endosomal sorting complex required for transport (ESCRT) pathway is a multifunctional pathway involved in cell physiological activities. While the majority of RNA viruses bearing L-domains are known to hijack the ESCRT pathway to complete the budding process, the budding of large and complex enveloped DNA viruses, especially iridoviruses, has been rarely investigated. In the present study, we use the tiger frog virus (TFV) as a model to investigate whether iridoviruses are released from host cells through the ESCRT pathway. Inhibition of class E proteins and auxiliary proteins (VPS4A, VPS4B, Tsg101, Alix, and Nedd4.1) reduces extracellular virion production, which preliminarily indicates that the ESCRT pathway is involved in TFV release. The respective interactions of TFV VP031L, VP065L, VP093L with Alix, Tsg101, Nedd4 suggest the underlying molecular mechanism by which TFV gets access to the ESCRT pathway. Co-depletion of Alix, Tsg101, and Nedd4.1 induces a significant reduction in extracellular virion production, which implies the functional redundancy of host factors in TFV budding. Those results are first observation that iridovirus gains access to ESCRT pathway through three ways of interactions between viral proteins and host proteins. Our study provides a better understanding of the budding mechanism of enveloped DNA viruses.

The cellular endosomal sorting complex required for transport (ESCRT) pathway is a multifunctional pathway involved in cell physiological activities^1^. The roles of the ESCRT pathway in eukaryotic cells include regulation of cargo-containing vesicles bud into endosomes to form multivesicular bodies (MVBs)^2^, cytokinesis^3^, exosome secretion^4^, and autophagy^5^. The ESCRT components (also known as class E proteins) comprise five distinct complexes, namely, ESCRT-0, -I, -II, -III, and AAA ATPase vacuolar protein sorting-4 (VPS4), as well as several auxiliary proteins, including apoptosis-linked-gene-2-interacting protein x (Alix) and Nedd4-like ubiquitin ligases^6^.

The assembly and release of enveloped viruses is a complex process that involves intricate interactions among the viral genome, viral proteins, and corresponding parasitized cellular factors. A newly formed virion bud is followed by a fission event that is required to overcome the energy barrier that the continuous cell membrane is broken and resealed to create discrete viral and cellular membranes^7^. Because numerous viruses do not encode their own membrane fission machinery, they hijack the ESCRT pathway to complete budding. Viruses encode a short motif that works at a very late stage during their life cycle, and is therefore named late domain (L-domain)^8^. The L-domain binds directly to ESCRT components as an upstream-acting factor to access the ESCRT pathway. To date, three types of L-domain have been described: P (T/S) AP, PPxY, and YPXnL (X = any amino acid, n = 1–3)^9^. The P (T/S) AP domain is the first L-domain to be identified in the p6 Gag protein of human immunodeficiency virus-1 (HIV-1) that is required for budding^10^ and has been shown to promote budding via direct interaction with tumor susceptibility gene 101 (Tsg101)^7^. The PPxY domain present in the structural proteins of vesicular stomatitis virus^11^, Ebola virus^12^, and Rous sarcoma virus (RSV)^13^ interacts with the WW domains present in Nedd4-like ubiquitin ligases^6^. The YPXnL domain functions by binding directly to Alix and is required for the budding of equine infectious anemia virus (EIAV)^14^ and Sendai virus^15^. Some viruses contain more than one L-domain in their respective proteins, but the functions of these domains in virus budding may not be equally important^16^,^17^. Until now, few studies have been conducted on the budding of enveloped DNA viruses, especially large and complex DNA viruses, compared with enveloped RNA viruses.

Iridoviruses are large and icosahedral enveloped DNA viruses that contain circularly permutated, terminally redundant, and double-stranded DNA genomes^18^. The family *Iridoviridae* has been subdivided into the following five genera: *Iridovirus, Chloriridovirus, Ranavirus, Lymphocystivirus,* and *Megalocystivirus*^19^. Members of the genus *Ranavirus* have been recognized as the major viral pathogens that infect amphibians, fishes, and reptiles^20^, resulting in huge economic losses in fish and frog aquaculture. In addition, members are lethal to certain endangered species, such as the Chinese giant salamander^21^. The tiger frog virus (TFV) is isolated from infected tadpoles of *Rana tigrina rugulosa*, causes high mortality rate among tiger frog tadpoles cultured in Southern China^22^. TFV is the first reported complete genome sequence in the genus *Ranavirus*, its genome comprises double-stranded DNA of 105,057 basepairs in length and is organized by 105 non-overlapping open reading frames (ORFs)^23^, sharing marked sequence identity with frog virus 3 (FV3), the type specie of the genus^24^. TFV infects a wide range of cell lines at 27 °C, including not only fish cell lines like fathead minnow (FHM) cells and zebrafish embryonic fibroblast (ZF4) cells^25^, but also mammalian cell lines, such as HepG2 cells^26^. To date, studies on the mechanisms of FV3 entry, viral transcription, and genome replication serve as a model of other *Iridoviridae* members, moreover, uptake of TFV into mammalian cells (HepG2) at 27 °C has been elucidated^26^. However, little is understood about the budding process.

In the present study, the role of class E proteins and ESCRT pathway in TFV release and the interaction mechanisms of class E proteins recruited to facilitate TFV budding are investigated. This study is the first to report that virus hijacks three ways of ESCRT pathway to complete virus budding. The findings of this work may provide novel insights into the development of anti-virus strategies.

## Results

### VPS4 is important for efficient TFV budding

**Figure 1:**
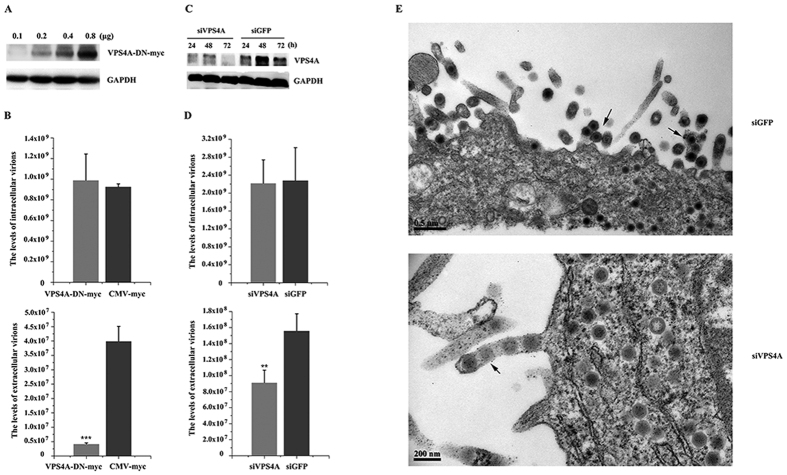
Production of the tiger frog virus (TFV) was inhibited by dominant-negative (DN) and RNA interference of VPS4A. (**A**) HepG2 cells were transfected with 0.1, 0.2, 0.4, or 0.8 μg of pVPS4A-DN-myc. Protein samples were collected and analyzed by Western blot, and GAPDH was included as control. (**B**) HepG2 cells were infected with TFV for 1 h after transfection with 0.8 μg of pVPS4A-DN-myc or pCMV-myc and then cultured for 66 h. Cells and supernatant were lysed to extract DNA. Samples were then subjected to absolute real-time quantitative PCR (qPCR) using major capsid protein (MCP) primers to quantify TFV genomic concentration. Statistical analysis was performed using Student’s *t*-test (0.05 < *p ≤ 0.1, 0.01 < **p ≤ 0.05, 0.01 ≤ ***p). (**C**) 100 nM of siRNA specific for VPS4A and control RNA (siGFP) were transfected into cells. The cells were collected from 24 h to 72 h and subjected to Western blot. (**D**) 100 nM siVPS4A or 100 nM siGFP were transfected into cells 6 h prior to infection with TFV for 1 h and then cultured for 66 h. Cells and supernatant were lysed to extract DNA. Samples were then subjected to qPCR using MCP primers to quantify TFV genomic concentration. Statistical analysis was performed using Student’s *t*-test (0.05 < *p ≤ 0.1, 0.01 < **p ≤ 0.05, 0.01 ≤ ***p). (**E**) EM analysis of HepG2 cells transfected with 100 nM siVPS4A or siGFP prior to infection with TFV for 66 h. Arrows indicated virions tethered to the plasma membrane.

**Figure 2:**
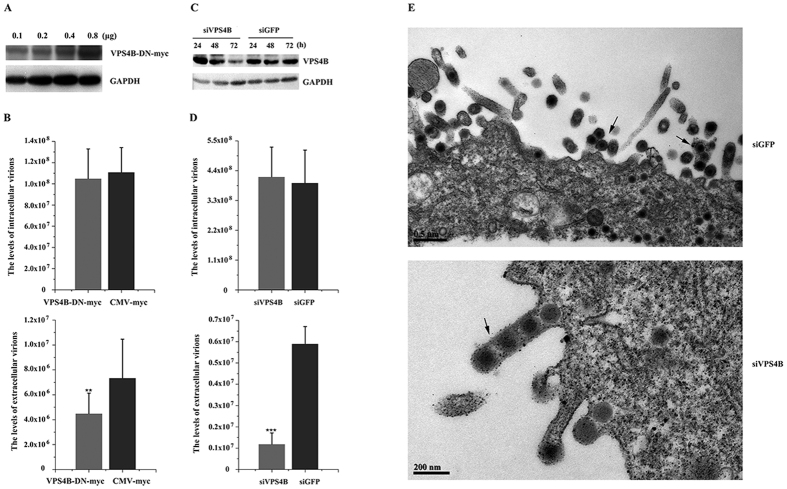
Production of TFV was inhibited by DN and RNA interference of VPS4B. (**A**) HepG2 cells were transfected with 0.1, 0.2, 0.4, or 0.8 μg of pVPS4B-DN-myc. Protein samples were collected and analyzed by Western blot, and GAPDH was included as control. (**B**) HepG2 cells were infected with TFV for 1 h after transfection with 0.8 μg of pVPS4B-DN-myc or pCMV-myc and then cultured for 66 h. Cells and supernatant were lysed to extract DNA. Samples were then subjected to qPCR using MCP primers to quantify TFV genomic concentration. Statistical analysis was performed using Student’s *t*-test (0.05 < *p ≤ 0.1, 0.01 < **p ≤ 0.05, 0.01 ≤ ***p). (**C**) 100 nM of siRNA specific for VPS4B and control RNA (siGFP) were transfected into cells. The cells were collected from 24 h to 72 h and subjected to Western blot. (**D**) 100 nM siVPS4B or 100 nM siGFP were transfected into cells 6 h prior to infection with TFV for 1 h and then cultured for 66 h. Cells and supernatant were lysed to extract DNA. Samples were then subjected to qPCR using MCP primers to quantify TFV genomic concentration. Statistical analysis was performed using Student’s t-test (0.05 < *p ≤ 0.1, 0.01 < **p ≤ 0.05, 0.01 ≤ ***p). (**E**) EM analysis of HepG2 cells transfected with 100 nM siVPS4B or siGFP prior to infection with TFV for 66 h. Arrows indicate virions tethered to the plasma membrane.

VPS4 is required for the budding of almost all viruses that are known to utilize the ESCRT pathway and appear to constitute the key machinery for budding^9^. Thus, ATPase activity-defective VPS4 is a useful tool to investigate the involvement of the ESCRT machinery in TFV budding. Mammals express two closely related VPS4 proteins (A and B), the functions of which are interchangeable in some contexts. VPS4 point mutant forms were used to verify the function of VPS4 in TFV budding. Figures 1A and 2A showed that the dominant-negative (DN) protein expression levels were dose-dependent with the amount of plasmids. Endogenous GAPDH levels were used as an internal loading control for Western blot analysis, so the highest plasmid amount was used in subsequent experiments. Based on absolute real-time quantitative PCR (qPCR) analysis, the intracellular virus genome equivalents (GEs) of producer cells transfected with the two VPS4 DN forms were 106.82% and 95% contrast with the CMV-myc control (Figs 1B and 2B), indicated that VPS4 DN forms function specifically in extracellular particle production. In contrast to CMV-myc, the VPS4A and VPS4B DN forms decreased extracellular particle production to 10.52% and 61.27%, respectively (Figs 1B and 2B). These results suggested that VPS4A and VPS4B were involved in TFV virus budding.

Overexpression of VPS4 point mutant forms act in a DN manner to decrease TFV particle production, but endogenous VPS4A and VPS4B are sufficient for budding^27^. Thus, the DN expression levels might not be high enough to block VPS4 function. Therefore, RNA interference was used to knock down endogenous VPS4 expression. Figures 1C and 2C showed that 100 nM siVPS4A and siVPS4B, not siGFP control, caused a severe reduction in the expression levels of endogenous VPS4 from 24 h to 72 h during the budding process. Thus, 100 nM siVPS4A and siVPS4B were used to investigate whether exhaustion of endogenous proteins by siRNAs would reduce the production of TFV (Figs 1D and 2D). Results showed that production levels of the extracellular TFV particles transfected with siVPS4A and siVPS4B decreased to 58.6% and 20.08%, respectively, compared with those of the siGFP control, although the production of the corresponding intracellular TFV GEs (97% and 105.55%) was not affected. The obtained results were consistent with data on DN mutants, which demonstrated that loss of VPS4 would decrease TFV budding.

Transmission electron microscopy was used to explore and visualize the TFV-infected cells. The intracellular unenveloped particles of the siGFP control were morphologically normal and displayed very few cell-associated virions and an abundance of released, mature, and enveloped virions were observed in the extracellular environment. Cells transfected with siVPS4A and siVPS4B caused virus arrest at a very late stage during assembly. During this stage, extensive clusters of immature particles accumulated at the cell membrane by long membrane stalks, although the intracellular unenveloped particles were morphologically normal (Figs 1E and 2E).

Taken together, the data demonstrated that VPS4 was important for TFV budding at a late stage of the virus life cycle and that TFV utilized the ESCRT pathway for budding.

### Tsg101, Alix, and Nedd4 are functionally involved in TFV budding

**Figure 3:**
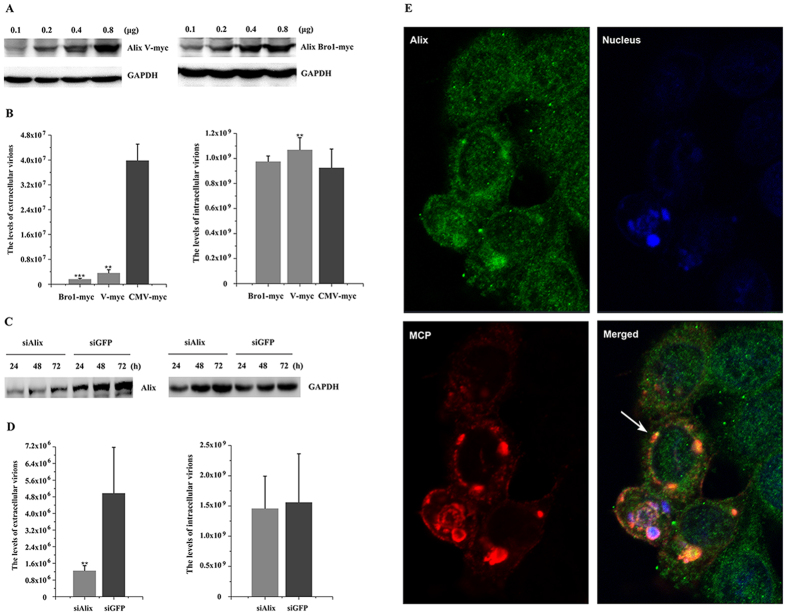
Production of TFV was inhibited by DN and RNA interference of Alix. (**A**) HepG2 cells were transfected with 0.1, 0.2, 0.4, or 0.8 μg of pAlix-V/Bro1 domain-myc. Protein samples were collected and analyzed by Western blot, and GAPDH was included as control. (**B**) HepG2 cells were infected with TFV for 1 h after transfection with 0.8 μg of pAlix-V/Bro1 domain-myc or pCMV-myc and then cultured for 66 h. Cells and supernatant were lysed to extract DNA. Samples were then subjected to qPCR using MCP primers to quantify TFV genomic concentration. Statistical analysis was performed using Student’s *t*-test (0.05 < *p ≤ 0.1, 0.01 < **p ≤ 0.05, 0.01 ≤ ***p). (**C**) 100 nM of siRNA specific for Alix and control RNA (siGFP) were transfected into cells. The cells were collected from 24 h to 72 h and then subjected to Western blot. (**D**) 100 nM siAlix or 100 nM siGFP were transfected into cells 6 h prior to infection with TFV for 1 h, then cultured for 66 h. Cells and supernatant were lysed to extract DNA. Samples were then subjected to qPCR using MCP primers to quantify TFV genomic concentration. Statistical analysis was performed using Student’s *t*-test (0.05 < *p ≤ 0.1, 0.01 < **p ≤ 0.05, 0.01≤***p). (**E**) *In vivo* confocal microscopy analysis was performed after HepG2 cells were infected with TFV for 1 h and cultured for 66 h. Cells were processed for immunostaining as described under “Materials and Methods”. Arrow indicates co-localization of TFV MCP and endogenous Alix.

**Figure 4:**
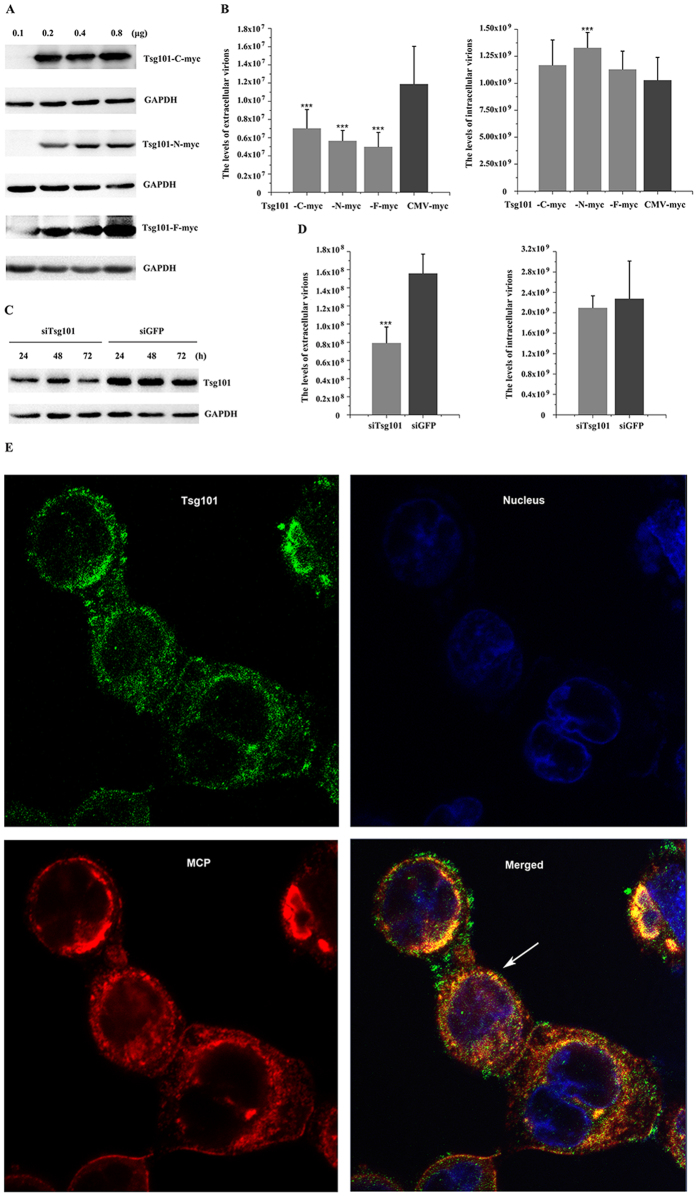
Production of TFV was inhibited by DN and RNA interference of Tsg101. (**A**) HepG2 cells were transfected with 0.1, 0.2, 0.4, or 0.8 μg of pTsg101-N/C/F-myc. Protein samples were collected and analyzed by Western blot, and GAPDH was included as control. (**B**) HepG2 cells were infected with TFV for 1 h after transfection with 0.8 μg of pTsg101-N/C/F-myc or pCMV-myc and then cultured for 66 h. Cells and supernatant were lysed to extract DNA. Samples were then subjected to qPCR using MCP primers to quantify TFV genomic concentration. Statistical analysis was performed using Student’s *t*-test (0.05 < *p ≤ 0.1, 0.01 < **p ≤ 0.05, 0.01 ≤ ***p). (**C**) 100 nM of siRNA specific for Tsg101 and control RNA (siGFP) were transfected into cells. The cells were collected from 24 h to 72 h and then subjected to Western blot. (**D**) 100 nM siTsg101 or 100 nM siGFP were transfected into the cells 6 h prior to infection with TFV for 1 h and then cultured for 66 h. Cells and supernatant were lysed to extract DNA. Samples were then subjected to qPCR using MCP primers to quantify TFV genomic concentration. Statistical analysis was performed using Student’s *t*-test (0.05 < *p ≤ 0.1, 0.01 < **p ≤ 0.05, 0.01 ≤ ***p). (**E**) *In vivo* confocal microscopy analysis was performed after HepG2 cells were infected with TFV for 1 h and cultured for 66 h. Cells were processed for immunostaining as described under “Materials and Methods”. Arrow indicates co-localization of TFV MCP and endogenous Tsg101.

Tsg101 is a component of the ESCRT-I complex^1^, Alix acts as a bridge between ESCRT-I and ESCRT-III^28^, and Nedd4 is involved in the ubiquitination of proteins to initiate sorting toward internal MVB vesicles^29^, and they bind directly to the L-domains, which are considered as the access to the ESCRT pathway^12^,^15^,^30^. The roles of Tsg101, Alix, and Nedd4 in TFV release were investigated using the analogous approaches described above. Alix and Tsg101 truncations expressed more proteins along with increasing amount of plasmids (Figs 3A and 4A). The highest plasmid concentrations were used for subsequent experiments. Figure 3B showed that production levels of intracellular virus GEs via overexpression of Alix-Bro1 domain and Alix-V domain were 105.42% and 115.11%, respectively, compared with that of the CMV-myc control, whereas the release of extracellular particles resulted in lower production levels of 4.23% and 9.14%, respectively. These results demonstrated that Alix-Bro1 domain and Alix-V domain were DN forms in TFV budding when overexpressed in HepG2 cells. Similarly, the efficiency of extracellular virus production was sensitive to the overexpression of the Tsg101-N terminal, Tsg101-C terminal, and Tsg101 full length (Tsg101-F). Production levels were reduced to 47.7%, 59.3%, and 42.2%, respectively, relative to the CMV-myc control. None of these reductions significantly affected the accumulation of intracellular virus GEs, because these GEs accumulated by 126%, 112%, and 107%, respectively, relative to the control (Fig. 4B). Thus, the Tsg101-N terminal, Tsg101-C terminal, and Tsg101-F were DN forms in TFV budding.

**Figure 5:**
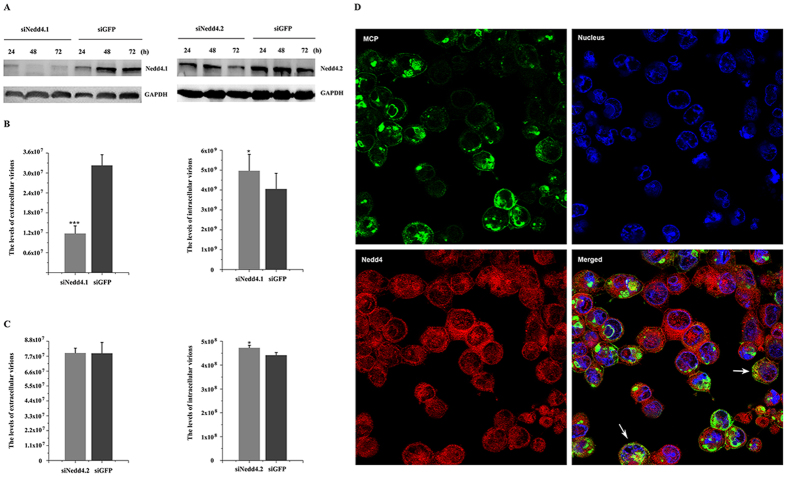
Production of TFV was inhibited by RNA interference of Nedd4.1. (**A**) 100 nM siRNA specific for Nedd4.1/Nedd4.2 and control RNA (siGFP) were transfected into cells. The cells were collected from 24 h to 72 h and then subjected to Western blot. (**B,C**) 100 nM siNedd4.1/Nedd4.2 or 100 nM siGFP were transfected into cells 6 h prior to infection with TFV for 1 h and then cultured for 66 h. Cells and supernatant were lysed to extract DNA. Samples were then subjected to qPCR using MCP primers to quantify TFV genomic concentration. Statistical analysis was performed using Student’s *t*-test (0.05 < *p ≤ 0.1, 0.01 < **p ≤ 0.05, 0.01 ≤ ***p). (**D**) *In vivo* confocal microscopy analysis was performed after HepG2 cells were infected with TFV for 1 h and cultured for 66 h. Cells were processed for immunostaining as described under “Materials and Methods”. Arrows indicate co-localization of TFV MCP and endogenous Nedd4.

Expression levels of Alix, Tsg101, and Nedd4 were down-regulated to study their effects on TFV budding. Nedd4 has two isoforms which are Nedd4.1 and Nedd4.2^31^. Thus, two individual RNA sequences corresponding to particular sequences in the two isoforms were designed such that they do not interfere with each other. Figures 3C, 4C, and 5A showed that 100 nM siAlix, siTsg101, siNedd4.1, and siNedd4.2, but not the siGFP control, markedly reduced the expression levels of endogenous target proteins from 24 h to 72 h during the budding process. Therefore, 100 nM siAlix, siTsg101, and siNedd4 were used to investigate whether exhaustion of these endogenous proteins by siRNA reduced the TFV production. Results showed that the extracellular TFV particle productions upon transfection with siAlix and siTsg101 decreased to 25.06% and 51%, respectively, relative to the siGFP control. Corresponding intracellular TFV GEs were 93.7% and 92.1% (Figs 3D and 4D). Surprisingly, extracellular particle production of cells transfected with siNedd4.1 was reduced to 36.5%, but production was almost invariant in cells transfected with siNedd4.2, in which intracellular virus GEs were 122% and 107%, respectively (Fig. 5B,C). The above mentioned results suggested that the Nedd4.1 isoform contributed to TFV budding in HepG2 cells. The data indicated that loss of Alix, Tsg101, and Nedd4.1 compromised viral budding.

*In vivo* confocal microscopy studies were conducted to confirm whether TFV recruited Alix, Tsg101, and Nedd4 to facilitate budding. TFV major capsid protein (MCP) was available as marker of the TFV particle. Alix was observed to be distributed throughout the cell and Tsg101 showed cytoplasmic staining with some enrichment near the nucleus. The overlay of the fluorescence patterns revealed extensive co-localization of TFV MCP with Alix and Tsg101 (Figs 3E and 4E). Nedd4 was expressed throughout the cytoplasm and nucleus and partly co-localized with TFV MCP in the cytoplasm, but not in the nucleus (Fig. 5D). The imaging data showed that Alix, Tsg101, and Nedd4 were recruited to the budding site to facilitate virus release.

Therefore, Alix, Tsg101, and Nedd4.1 were functionally involved in TFV budding in HepG2 cells.

### TFV L-domains contained proteins interact with Alix, Tsg101, and Nedd4

Bioinformatics analysis of the TFV sequence revealed more than 20 proteins containing potential L-domains. All kinds of L-domains were found in different viral proteins, even in one viral protein (data unpublished). Thus, experiments were performed to determine whether the viral proteins containing putative L-domains immunoprecipitate with endogenous corresponding host proteins to investigate the mechanisms by which TFV acquires access to the host ESCRT pathway.

**Figure 6:**
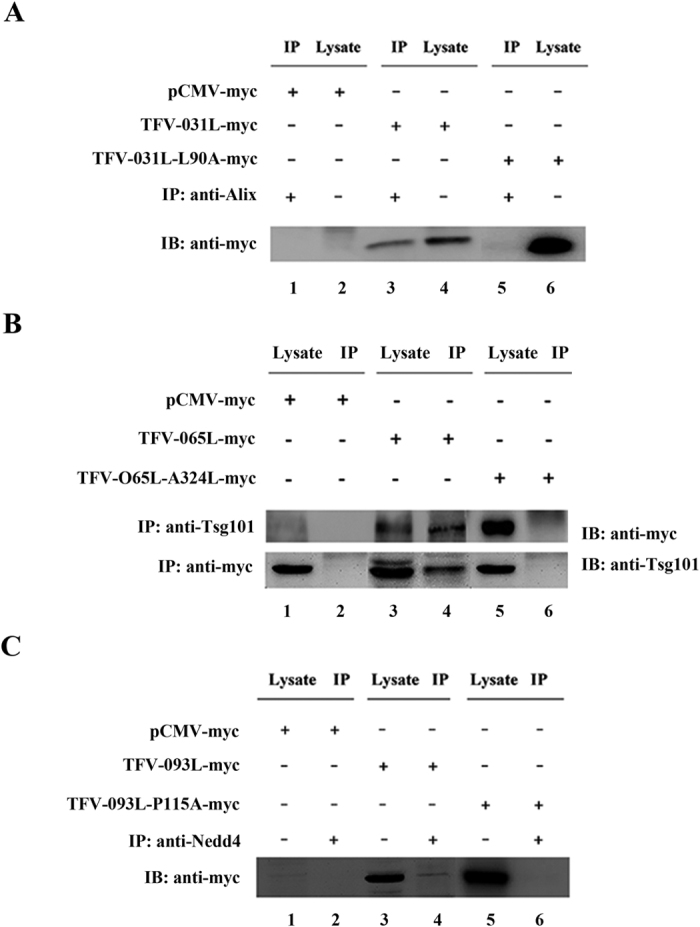
Identification of the protein–protein interaction between TFV protein containing putative L-domain and corresponding host protein using immunoprecipitation (IP). (**A**) IP of VP-031L and Alix using anti-Alix antibody. Hela cells were transfected with pVP-031L-myc, pVP-031L-L90A-myc, or an empty vector pCMV-myc. Cells were processed for IP as described under “Materials and Methods”. Anti-myc antibody was used as the primary antibody for IP. (**B**) IP of VP-065L and Tsg101 using anti-myc antibody and anti-Tsg101 antibody. Hela cells were transfected with pVP-065L-myc, pVP-065L-A342L-myc, or an empty vector pCMV-myc. Cells were processed for IP as described under “Materials and Methods”. Anti-Tsg101 antibody and anti-myc antibody were used as the primary antibodies for IP. (**C**) IP of VP-093L and Nedd4 using anti-Nedd4 antibody. Hela cells were transfected with pVP-0931L-myc, pVP-093L-P115A-myc, or an empty vector pCMV-myc. Cells were processed for IP as described under “Materials and Methods”. Anti-myc antibody was used as the primary antibody for IP.

According to the bioinformatics analysis, TFV VP031L, VP065L, and VP093L contained the YPXnL, ASAP, and PPxY domain, respectively, but their functions in the virus life cycle remain ambiguous. The results showed that VP031L was detected after the anti-Alix antibody was used to immunoprecipitate endogenous Alix (Fig. 6A, line 3), and no band was detected when pCMV-myc was transfected as control vector (Fig. 6A, line 1). Similarly, VP065L was detected after the anti-Tsg101 antibody was used to immunoprecipitate endogenous Tsg101 (Fig. 6B, line 4, top), no band was detected when pCMV-myc was transfected as control vector (Fig. 6B, line 2, top), and Tsg101 protein was also detected after the anti-myc antibody was used to immunoprecipitate transfected pVP065L-myc (Fig. 6B, line 4, bottom). In addition, VP093L was detected after anti-Nedd4 antibody was used to immunoprecipitate endogenous Nedd4 (Fig. 6C, line 4), and no band was detected when pCMV-myc was transfected as the control vector (Fig. 6C, line 2). The function of the L-domain in these interactions, was confirmed by replacing one amino acid of L-domain with an irrelevant one. Contrary to the wild-type (WT) L-domains, VP031L-L90A was not detected after the anti-Alix antibody was used to immunoprecipitate endogenous Alix (Fig. 6A, line 5). Similarly, VP065L-A342L was not detected after the anti-Tsg101 antibody was used to immunoprecipitate endogenous Tsg101, and no band was detected after the anti-myc antibody was used to immunoprecipitate transfected pVP065L-A342L-myc (Fig. 6B, line 6). In addition, VP093L-P115A was not detected after the anti-Nedd4 antibody was used to immunoprecipitate endogenous Nedd4 (Fig. 6C, line 6).

More than 20 sets of immunoprecipitation experiments were conducted to test whether the viral proteins containing putative L-domains interacted with endogenous corresponding host proteins (data unshown). Three viral proteins were found to directly interact with Alix, Tsg101, and Nedd4. Moreover, three sets of immunoprecipitation experiments using point mutant L-domains showed that the L-domain played roles in these interactions. These binding interactions represent the mechanism by which TFV recruits ESCRT pathway for budding.

### Alix, Tsg101, and Nedd4.1 are functionally redundant in TFV budding

The majority of viruses express proteins containing one or two L-domains, and the interactions with corresponding host proteins are sufficient for budding^16^,^32^. In the present study, all three types of L-domains were present in TFV proteins, and these domains recruited downstream host factors. Additionally, reduced activity of Alix, Tsg101, and Nedd4.1 led to modest inhibitions in TFV budding, so we tested whether these L-domain interacted proteins were functionally redundant. We analyzed the effects of simultaneous depletion of multiple proteins with various combinations.

**Figure 7:**
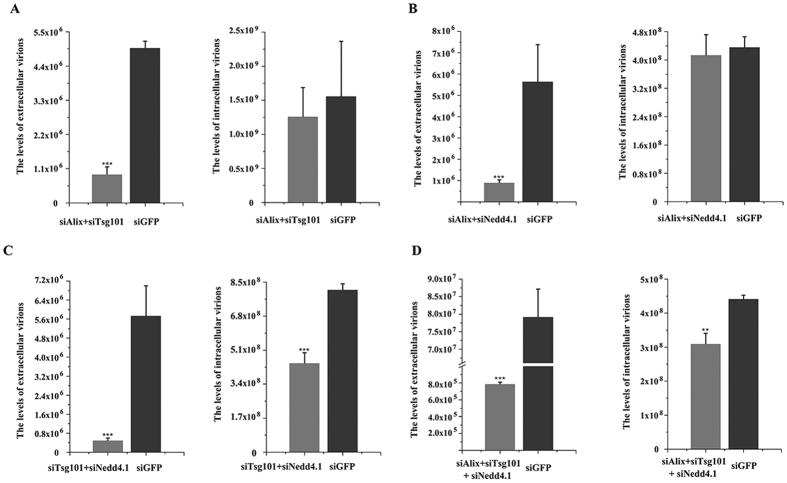
Production of TFV was more severely inhibited by co-depletion of Alix, Tsg101, and Nedd4.1. (**A**) Co-depletion of 100 nM siAlix and 100 nM siTsg101 or 100 nM siGFP were transfected into cells 6 h prior to infection with TFV for 1 h, and then cells were cultured for 66 h. Cells and supernatant were lysed to extract DNA. Samples were then subjected to qPCR using MCP primers to quantify TFV genomic concentration. (**B**) Co-depletion of 100 nM siAlix and 100 nM siNedd4.1 compared with 100 nM siGFP. (**C**) Co-depletion of 100 nM siTsg101 and 100 nM siNedd4.1 compared with 100 nM siGFP. (**D**) Co-depletion of 100 nM siTsg101, 100 nM siNedd4.1, and 100 nM siAlix compared with 100 nM siGFP. Statistical analysis was performed using Student’s *t*-test (0.05 < *p ≤ 0.1, 0.01 < **p ≤ 0.05, 0.01 ≤ ***p).

Co-depletion of Alix and Tsg101, Alix and Nedd4.1, or Tsg101 and Nedd4.1 using siRNA resulted in more significant reduction compared with single depletion, that is, 18.41%, 15.95%, and 8.77% relative to the siGFP control, respectively (Fig. 7A–C). Surprisingly, co-depletion of Alix, Tsg101, and Nedd4.1 markedly decreased TFV budding, with production reduced to 1% compared with siGFP control (Fig. 7D). Intracellular virus GEs decreased to 80.88%, 95.08%, 54.77%, and 70.14% in co-depletions (Fig. 7A–D), but extracellular particle production decreased considerably.

Thus, Alix, Tsg101, and Nedd4.1 were recruited in parallel by viral proteins and contributed to complete the TFV budding. Such functional redundancy may allow for the loss of one or two interactions between L-domain contained viral proteins or corresponding host proteins.

## Discussion

**Figure 8:**
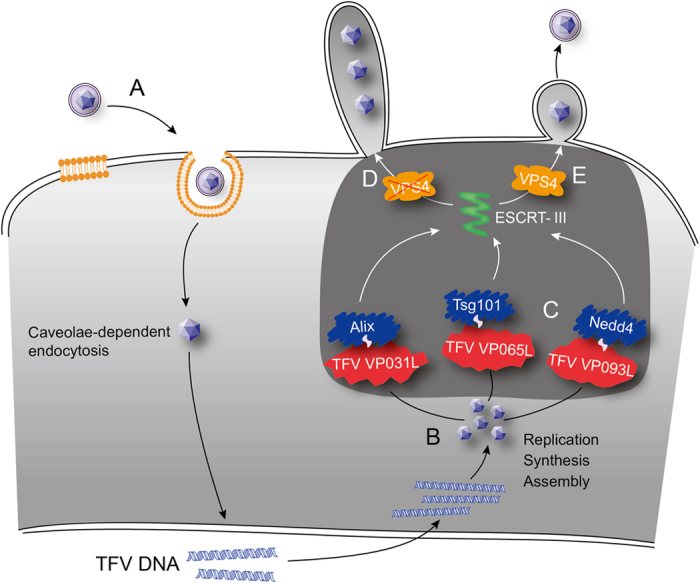
Model of cellular budding of TFV. (**A**) TFV internalizes into the host cells via caveolae. (**B**) TFV replicates and assembles at the nucleus and cytoplasm. (**C**) TFV gains access to ESCRT pathway through three ways of interactions between viral proteins and host proteins. (**D**) Loss of VPS4 leads to defective budding to form long stalk containing more than one virus particle tethered to membrane budding. (**E**) VPS4 functions in budding and formation of mature enveloped virus particles.

Iridoviruses are large and complex enveloped DNA viruses, but little information is available on their virus budding mechanism. In this study, TFV relied on the network of ESCRT proteins to complete virus budding. The present work was the first to report an example of the budding mechanism of an iridovirus. In addition, results showed the first observation that three approaches were used to access the pathway, and all these approaches contributed to complete virus budding (Fig. 8).

VPS4 is a member of the AAA ATPase family, which is an essential protein for MVB formation. This protein powers the pathway by converting the energy of ATP hydrolysis into mechanical work^6^ and dissociates ESCRT complexes from the MVB membrane, facilitating their recycling for further rounds of vesicle formation^33^. Previous studies have shown that VPS4 is required for efficient budding of many RNA viruses and Herpes simplex virus-I (HSV-I) in using DN VPS4^9^,^34^,^35^. We first detected the role of VPS4 in TFV budding considering that it is a pivotal factor in virus budding. Disruption of VPS4A or VPS4B expression, either by defective forms overexpressed by transit transfection or down-regulated by siRNA silencing, caused the marked effect of TFV budding. These results indicated that VPS4A and VPS4B were clearly required for efficient budding of TFV. Considering that the two isoforms of VPS4 both play roles in budding, it is not curious that virus release is not absolutely blocked by defective VPS4A or VPA4B. Thus, the effect of co-depletion needs further investigation.

Enveloped viruses hijack the ESCRT pathway through interactions between viral proteins bearing L-domains and class E proteins. In this report, bindings between TFV VP031L and Alix, VP065L and Tsg101, VP093L and Nedd4 were presented, while VP031L and VP093L were too small to isolate the target proteins, which were much larger than itself from extract, and L-domain mutant viral proteins did not interact with corresponding host proteins. These results further showed that interactions with class E proteins to L-domain contained TFV proteins appeared to be the access to the ESCRT machinery and play roles in TFV budding. Notably, ASAP domain in VP065L was PSAP-like L-domain, which was first identified in arenavirus, ASAP was not observed to significantly contribute to arenavirus budding, however, the motif in TFV VP065L obviously interacted with Tsg101 and could function instead of PSAP^35^,^36^.

Tsg101-N terminal was the binding domain that interacted with the P(T/S)AP motif. Overexpression of this terminal inhibited viral budding by competitively binding with the PTAP motif and inactivating endogenous Tsg101, such that the Tsg101-N terminal specifically disrupted virus budding in a P(T/S)AP L-domain dependent manner^17^,^37^. The Tsg101-C terminal was the binding site for the other subunits of the ESCRT-I^38^, and its overexpression severely inhibited HIV-1 and RSV budding^39^. Overexpression of Tsg101-F inhibited budding in HIV-1, feline immunodeficiency virus, and Lassa virus^27^,^38^,^40^. Transfection of siTsg101 caused a marked reduction in the released particles in hepatitis E virus, Lassa virus, and bluetongue virus^27^,^30^,^41^. Alix-Bro1 domain served as the binding site for the ESCRT-III component CHMP4, Alix-V domain served as the binding site for YPXnL motifs^42^. Thus, Alix DN mutants suppressed the egress of the EIAV, HIV-1, HTLV-1, and Mopeia virus VLP production^42^,^43^,^44^. SiRNA silencing of Alix and Tsg101 or the overexpression of Tsg101-F, Tsg101-N terminal, Tsg101-C terminal, Alix-Bro1 domain, and Alix-V domain partially inhibited the release of TFV. These results verified that Tsg101 and Alix were involved in budding. Nedd4 is an active E3 ubiquitin ligase that has two isoforms, which were both down-regulated in the current study using siRNAs, results showed that Nedd4.1 had a role in TFV budding. Because of the knockdown effect by siNedd4.2 is weaker than siNedd4.1, determining whether Nedd4.2 affects budding requires further studies. In addition, co-localization of the viral MCP protein with Alix, Tsg101, and Nedd4.1 stated that cellular ESCRT machinery was recruited to TFV budding site. Taken together, the interactions between viral proteins bearing L-domains and their corresponding class E proteins were important for TFV budding, and the involved host factors all had roles in TFV budding.

The majority of RNA viruses express one or two L-domains in a specific protein, and the use of two distinct L-domains have equivalent and therefore redundant functions in virus release^45^. The HTLV-1 Gag protein contained the PPPYEPTAP motif and recruited both Tsg101 and Nedd4 via its PTAP and PPxY L-domains to efficiently exit from the cell^16^. The HIV-1 Gag protein contained the PTAP and YPXnL domains and blocked PTAP-Tsg101 or YPXnL-Alix interactions both affected HIV-1 release^14^. The case with DNA virus was apparently more complex, because HSV-1 encoded all three classes of L-domains in several structural proteins and required functional ESCRT III complex and VPS4 but independent of Tsg101 and Alix^46^. The obtained data indicated that numerous L-domains existed in different viral proteins in the mature TFV virion. Experimental results show that three viral proteins bearing L-domains interacted with corresponding host factors. The high frequency of L-domain may signify the importance of engaging host factors for the release of nascent viruses and functional redundancy of different proteins in the TFV life cycle. As previously mentioned, inhibition of one of these host factors resulted in modest decrease in virus production, reflecting insufficient protein depletion and/or functional redundancy. Our siRNA protocols almost completely depleted the target proteins, so all possible pairwise knockdowns of Alix, Tsg101, and Nedd4.1 were tested. Compared with single depletion, synergistic inhibitions of TFV budding were observed in all cases. The strongest budding defects were induced upon simultaneous depletion of endogenous Alix, Tsg101, and Nedd4.1, in which viral budding was nearly completely blocked. These results indicated that a number of parallel mechanisms by which TFV could access the ESCRT machinery are redundant; any of these approaches could, to some extent, be sufficient for virus budding. In addition, three L-domains were located in different viral proteins, indicating that defects in one or two viral proteins or loss of any relevant factors in host cells could not completely block TFV propagation and may be associated with broad range of cell lines that allow TFV infection^40^.

The amounts of intracellular viral GEs were monitored during our experiments to verify that the reduction in released virus particles was not due to the failure to replicate virus in host cells. The intracellular producing virus GEs were not markedly decreased by the overexpression of ESCRT DN mutants and the silencing of class E proteins by siRNA. The results showed a special case in which single-depletion of Tsg101 led to a small reduction in intracellular particles, and co-depletion of Tsg101 and other auxiliary proteins resulted in more remarkable decrease in intracellular virus GEs. Tsg101 is a component of ESCRT-I^47^, which is an essential cellular complex in the ESCRT pathway^30^, so its down-regulation using siRNA might affect the functional integrity of the ESCRT pathway, whether the loss of Tsg101 affects TFV replication should be investigated further. The sharp reduction in the amount of nascent virions was largely affected by the budding process because the decrease in intracellular virus GEs was considerably lower than the decrease in extracellular amount.

Viruses employ the same pathway to complete budding, but the cellular locations at which the viral proteins assembled are varied. In some cases, immature virions bud across the plasma membrane into the extracellular space^48^. However, in other cases, nascent virions bud into the lumen of cellular organelles, with luminal fused with plasma membrane to release virions to extracellular environment^49^,^50^. Previous studies have shown that viruses that rely on the function of Tsg101 or Alix mainly bud from the plasma membrane^8^,^51^,^52^, whereas other viruses appear to bud into ER/Golgi/endosomal structures^53^,^54^. The plasma membrane was inferred to be the TFV budding site, given that TFV relied on Tsg101 and Alix, and transmission electron microscopy results implied that immature virions in the intracellular plasma membrane periphery were unenveloped. However, further investigation is required.

## Materials and Methods

### Cell lines and antibodies

*Homo sapiens* HepG2 (ATCC HB-8065) and Hela (ATCC CCL-2) cells were cultured as a monolayer at 37 °C in complete Dulbecco’s modified Eagle’s medium (Gibco, USA) supplemented with 10% (vol/vol) fetal bovine serum (FBS) (Gibco, USA) under a humidified atmosphere containing 5% CO_2_. Fathead minnow (FHM) cells (ATCC CCL-42) were grown in M199 medium supplemented with (Gibco, USA) 10% (vol/vol) FBS at 27 °C.

Rabbit and mouse polyclonal antisera against TFV MCP were previously generated in our laboratory. Rabbit monoclonal antibody against the MYC tag and anti-Nedd4 antibody was purchased from Abcam (UK). Rabbit monoclonal anti-GAPDH antibody was obtained from CST (BRD). Mouse monoclonal anti-Tsg101, anti-Alix, anti-VPS4B, and rabbit polyclonal anti-VPS4 antibody were acquired from Santa Cruz (USA). Anti-rabbit/mouse IgG (H+L)-HRP conjugates were purchased from Promega (USA). All antibodies were used according to the manufacturers’ instructions.

### Viral infection

TFV was originally isolated from diseased tiger frog (*Rana tigrina rugulosa*) tadpoles in Naihai, Guangdong, China and was preserved in our laboratory. TFV virus stock was generated using FHM cells inoculated with TFV suspension for 5 d and harvested when the cytopathic effect was observed. Virions were released by three freeze–thaw cycles and clarified using 0.45 μm filter. HepG2 cells were infected with the virions at a multiplicity of infection of 10.

### Plasmid construction and transient transfection

The plasmids used for the expression of the viral proteins were based on the sequences of TFV strain (AF389451.1)^23^. The plasmids VP031L-myc, VP065L-myc, and VP093L-myc were amplified using primers presented in Table 1.

Tsg101, VPS4A, and VPS4B coding sequences were amplified via reverse transcription (RT)-PCR using a total RNA extract from HepG2 cells (primers in Table 1), and subsequently subcloned into the plasmid vector to generate the recombinant plasmids Tsg101-myc, VPS4A-myc, and VPS4B-myc. Plasmid Alix-flag was purchased from Genecopoeia (China). The DN mutants of VPS4 were active site glutamate point mutated to glutamine to render them ATPase-defective^55^. Plasmids encoding VPS4A-E228Q and VPS4B-E235Q^56^ were generated using pVPS4A-myc and pVPS4B-myc as templates following the protocol of site-directed gene mutagenesis kit (Beyotime, China) (primers in Table 1). The sequences of mutant regions were confirmed by DNA sequencing (MegAlign). DN constructs of Alix were truncations of wild type (WT) Bro1 domain (residues 1–358) and WT V domain (residues 362–702)^14^. The Tsg101-N terminal (residues 1–238)^37^, Tsg101-C terminal (residues 239–401)^39^, and Tsg101-F were considered Tsg101 dominant forms^38^. Thus, the truncations were generated via PCR using pAlix-flag and pTsg101-myc as templates (primers in Table 1). The mutants of VP031L, VP065L, and VP093L were one amino acid of L-domain point mutated to an irrelevant one. Plasmids encoding VP031L-L90A, VP065L-A342L and VP093-P115A were generated using pVP031-myc, pVP065-myc, and pVP093L-myc as templates following the protocol of site-directed gene mutagenesis kit (Beyotime, China) (primers in Table 1). The sequences of mutant regions were confirmed by DNA sequencing (MegAlign). Transient transfections of plasmids into Hela and HepG2 cells were performed using Lipofectamine^TM^ 2000 (Invitrogen, USA) reagent according to the manufacturer’s protocol.

### RNA interference

To knock down cells of target proteins, siRNAs (Ribobio, China) targeting the proteins were synthesized and verified to be effective, since they decreased protein levels of the targets (sequences in Table 1). The siRNA against green fluorescent protein (GFP) was synthesized as negative control. siRNAs were transfected into cells at a final concentration of 100 nM using Hiperfect transfection reagent (Qiagen, BRD).

### Absolute real-time qPCR

HepG2 cells were plated overnight at a density of 1.5 × 10^5^ cells/well or 1 × 10^5^ cells/well in a 24-well culture plate. Then, 0.8 μg of WT/DN plasmid or 100 nM siRNA was transfected into cells 6 h prior to infection with TFV for 1 h. The cells were washed thrice with sodium citrate buffer. Then, fresh medium was added to the cells, and the cells were cultured for 66 h. The supernatant of the cells was clarified from the cell debris by centrifugation (3,000 rpm, 5 min). The experiment was performed in triplicate. DNA was extracted from the supernatant and cell by absolute real-time qPCR (primer sequences in Table 1) analysis using DNeasy Blood and Tissue Kit (Qiagen, BRD) according to the manufacturer’s instructions. Levels of TFV GE were determined using Light Cycler 480 (Roche, CH). qPCR were carried out in triplicate per sample.

### Electron Microscopy

HepG2 cells were transfected with 100 nM siVPS4A, siVPS4B, and siGFP. After 6 h, the cells were infected with TFV for 66 h before analysis. Cells were fixed, dehydrated, embedded, and sectioned for electron microscopy (FEI, USA) using standard methods.

### Immunoprecipitation (IP)

Hela cells were transfected with recombinant plasmids VP031L-myc, VP031L-L90A-myc, VP065L-myc, VP065L-A342L-myc, VP093L-myc, VP-093L-P115A-myc. After 48 h, the cells were lysed on ice with 1 ml of cell lysis buffer for Western blot analysis and IP (Beyotime, China) for 30 min. The cell lysates were then centrifuged at 12 000 rpm for 10 min. IP was performed according to the manufacturer’s instruction (Dynabeads protein immunoprecipitation kit; Life Technologies, USA). Proteins bound to the beads were released by boiling in 5× SDS loading buffer for 10 min. The samples were analyzed by Western blot.

### Western Blot

Cells transfected with siRNAs and DN plasmids were lysed on ice with cell lysis buffer, protein concentrations were determined with bicinchoninic acid protein assay kit (Beyotime, China). Equivalent amounts of total protein were boiled in 5× SDS sample loading buffer for 10 min, and IP samples were subjected to 12% SDS-PAGE. The WB procedure was performed as previously described^57^.

### Immunofluorescence labeling and confocal microscopy

HepG2 cells were seeded at coverslips and infected with TFV virus. The cells were washed with sodium citrate buffer 1 h post-infection to remove the unabsorbed virus, and the culture was replaced with fresh medium. The cells were incubated at 27 °C for a specified time, following procedure was performed as previously described^57^. The coverslips were mounted using Prolong Antifade Mountant (Life Technology) at room temperature overnight. Samples were viewed under a confocal microscope (LSM510; Zeiss, GER) equipped with 555 nm/488 nm argon-krypton and 543 nm helium-neon lasers.

### Statistical analysis

qPCR was performed in triplicate per sample. Data were analyzed using Roche Abs Quant/2nd Derivative Max, followed by an independent sample *t*-test to determine the statistical significance between controls and the experimental groups. Statistical significance was accepted at 0.05 < *p ≤ 0.1, 0.01 < **p ≤ 0.05, and 0.01 ≤ ***p.

## Additional Information

**How to cite this article**: Mi, S. *et al.* Budding of Tiger Frog Virus (an Iridovirus) from HepG2 Cells via Three Ways Recruits the ESCRT Pathway. *Sci. Rep.* **6**, 26581; doi: 10.1038/srep26581 (2016).

## Figures and Tables

**Table 1:** Summary of sequences used in this study.

Purpose	Sequences (5′-3′)^a^,^b^
Mammalian cell	
Expression vector	
pVP-031L-myc	CGGAATTCGGCGTTGTATGCGCTCCGGG
pVP-065L-myc	GAAGATCTCTTCCAGGGGCATGACTATCGAGG
pVP-093L-myc	CGGAATTCGGTACCTAAACGCGTTAGCCAAGG
pTsg101-myc	GGAATTCCGATGGCGGTGTCGG
pVPS4A-myc	GGAATTCCGATGACAACGTCAACCCTC
pVPS4B-myc	GGAATTCCGATGTCATCCACTTCGCC
pVPS4A-E228Q-myc	CCATCATCTTCATCGATCAGGTGGATTCCCTCTGC
pVPS4B-E235Q-myc	CCATTATCTTCATTGATCAGATTGATTCTCTCTGTGG
pAlix-Bro1 domain-myc	GGAATTCGGATGGCGACATTCATCTC
pAlix-V domain-myc	GGAATTCGGTCAGTACAGCAGTCTTTGG
pTsg101-N-myc	GGAATTCCGATGGCGGTGTCGG
pTsg101-C-myc	GGAATTCCGCTCATCTCTGCGGTC
pVP-031L-L90A-myc	GTTATCCAGCAGTAGCATTTCGTTCTCTC
pVP-065L-A342L-myc	CAGCACGCGTCCGCCTTAGAGG
pVP-093L-P115A-myc	GTCACACCATGGCCCCCGACT
RNA interfering	
siVPS4A	CCGAGAAGCUGAAGGAUUATT^58^
siVPS4B	CCAAAGAAGCACUGAAAGATT^58^
siAlix	GCCGCUGGUGAAGUUCAUCTT^44^
siTsg101	CAGTTTATCATTCAAGTGTAA^59^
siNedd4.1	TAGAGCCTGGCTGGGTTGTTTTG^60^
siNedd4.2	AACCACAACACAAAGTCACAG^60^
qPCR	
TFV-MCP	TCGCTGGTGGAGCCCTGGTA

^a^Underlined portion indicates restriction endonuclease cleavage sites.

^b^Bold portion indicates point mutant sites.
